# *LaueMatching*: an approach for rapid and robust indexing of Laue diffraction patterns

**DOI:** 10.1107/S1600576726001196

**Published:** 2026-03-11

**Authors:** Hemant Sharma, Dina Sheyfer, Ross Harder, Jonathan Z. Tischler

**Affiliations:** a Advanced Photon Source Argonne National Laboratory Lemont IL60439 USA; DESY, Hamburg, Germany

**Keywords:** Laue microdiffraction, indexing, image processing, orientation refinement, pattern correlation

## Abstract

Traditional Laue diffraction pattern indexing often struggles with noisy data, weak signals, peak overlap and missing reflections, particularly from complex or deformed microstructures. Here, we introduce *LaueMatching*, a high-throughput indexing algorithm designed to overcome these limitations. *LaueMatching* utilizes a fundamentally different approach based on direct pattern correlation: experimentally pre-processed images are compared against a comprehensive pre-computed library of simulated diffraction patterns corresponding to a dense grid of possible orientations. This approach bypasses the need for explicit peak identification and fitting, steps that are often a failure point for traditional methods. The algorithm rapidly and robustly indexes multiple crystallographic orientations and crystal systems simultaneously, even from challenging patterns. *LaueMatching*’s effectiveness and accuracy have been rigorously tested and validated on diverse experimental (Ni, Al, EuAl_2_O_4_) and simulated diffraction patterns, demonstrating high-fidelity orientation refinement. Code to implement this approach on both CPU and GPU resources can be downloaded from https://github.com/AdvancedPhotonSource/LaueMatching.

## Abstract 2

This paper introduces *LaueMatching*, a rapid and robust algorithm for indexing Laue diffraction patterns using a pre-computed simulation library. It employs pattern-correlation matching, overcoming challenges like weak signals, missing peaks and highly distorted peaks from complex microstructures which are often faced by traditional peak-finding methods.

## 1. Introduction

Materials science research relies heavily on understanding the microstructure of materials across varying length scales, from features down to the nanometre scale to millimetres and larger. The Laue microdiffraction technique occupies a unique position amongst the wide variety of techniques used to study the microstructure of materials. It provides non-destructive bulk information about the microstructure (crystallographic orientation and strain information) with sub-micrometre spatial resolution and 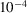 absolute strain resolution (Liu *et al.*, 2004 ▸; Chung & Ice, 1999 ▸; Ice & Larson, 2000 ▸). A detailed overview of the multiple facilities implementing this technique is provided by Barabash & Ice (2014 ▸). Laue microdiffraction works by rastering a sub-micrometre sized polychromatic X-ray beam across a sample, with the third dimension (depth resolution) coming from differential or coded aperture techniques (Tamura *et al.*, 2002 ▸; Larson & Levine, 2013 ▸; Gürsoy *et al.*, 2022 ▸).

Traditional techniques for determining orientation and strains from a Laue diffraction pattern involve detecting the position of diffraction peaks in the pattern, indexing the diffraction peaks to orientations, and refining the crystal structure for each position and depth (Tischler, 2014 ▸; Liu *et al.*, 2004 ▸; Barabash *et al.*, 2001 ▸; Plancher *et al.*, 2016 ▸; Zhang *et al.*, 2017 ▸, 2023 ▸; Petit *et al.*, 2015 ▸; Dejoie & Tamura, 2020 ▸; Li *et al.*, 2020 ▸; Vasilev *et al.*, 2023 ▸; Tamura, 2014 ▸). Recent improvements in data processing for Laue microdiffraction involve machine learning techniques such as LaueNN (Purushottam Raj Purohit *et al.*, 2022 ▸; Kirstein *et al.*, 2023 ▸), dictionary-based searches (Gupta & Agnew, 2009 ▸; Seret *et al.*, 2022 ▸) and zone-axis-assisted indexing (Kou & Chen, 2024 ▸). In addition to Laue microdiffraction, dictionary-based approaches and pattern matching have been successfully used to index diffraction patterns in applications such as electron backscatter diffraction (Singh & De Graef, 2016 ▸) and optical microscopy (Wittwer & Seita, 2022 ▸). Gevorkov *et al.* (2020 ▸) have developed an orientation-envelope-based indexing approach. All these methods rely on accurately determining the position of diffraction peaks to compute the angles between them, which can be challenging for heavily deformed materials. Another approach, recently proposed by Huang *et al.* (2023 ▸), avoids the peak-searching step but requires the user to manually select diffraction peaks to kick-start the orientation search. While these advances have improved indexing, a robust fully automated method that does not rely on finding and fitting well defined diffraction peaks, especially for patterns with numerous overlapping grains or weak signals, remains a critical need.

The methods described above struggle if patterns consist of diffraction signals from a large number of orientations, if the experimental configuration and crystal structure result in only a small number of diffraction peaks, if the number of missing peaks is high, or if the crystal structure is not well known. This paper introduces *LaueMatching*, a novel method that indexes Laue patterns through direct correlation matching against a comprehensive simulated pattern library, which inherently overcomes many of the limitations of methods that rely on precise peak fitting and centroiding. The bulk of the computational work (computing the library of diffraction patterns) is done *a priori*, leading to extremely high computational performance in orientation determination. Pre-computed diffraction patterns are stored and can be used repeatedly on diffraction data with the same experimental configuration. The method works well even for cases where the crystal structure is not well known. While a candidate crystal structure (lattice parameter and space group) is required to generate the simulation library, *LaueMatching* can be used to test multiple candidate structures against an experimental pattern; the structure yielding the highest figure of merit is the most probable match. Although developed here for Laue microdiffraction, the underlying principle of indexing via correlation against a comprehensive simulated library holds potential applicability to other diffraction-pattern indexing challenges where traditional feature extraction is difficult, *e.g.* diffraction from deformed materials and weak scattering signals. The experimental Laue geometry and forward-simulation model are described in Section 2. The workflow is detailed in Section 3. The robustness of *LaueMatching* is demonstrated using multiple simulated and experimental diffraction patterns in Section 4.

*LaueMatching* has been implemented as an application written in C and CUDA with Python bindings. Available to run on both CPU (on Linux and MacOS) and GPU (on NVIDIA GPUs) resources, the code can be downloaded from https://github.com/AdvancedPhotonSource/LaueMatching.

## 2. Forward simulations and Laue geometry

This section describes the geometry of the Laue microdiffraction setup and the forward-simulation model used to compute the diffraction-pattern library.

A key aspect of polychromatic ‘white-beam’ Laue diffraction is that reflections are defined by a combination of scattering angle and the specific X-ray energy that satisfies the Bragg condition, forming a locus of possible diffraction events in angle–energy space. Our forward-simulation model explicitly accounts for this. For each candidate orientation and each allowed *hkl* reflection, the model calculates both the corresponding pixel position on the detector and the required energy, *E*. This list of potential reflections is then filtered, retaining only the pixel positions for which *E* falls within the known experimental energy range (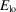, 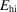). The simulation library therefore stores a set of 2D detector coordinates that implicitly represents the valid angle–energy combinations for each orientation.

**Figure 1:**
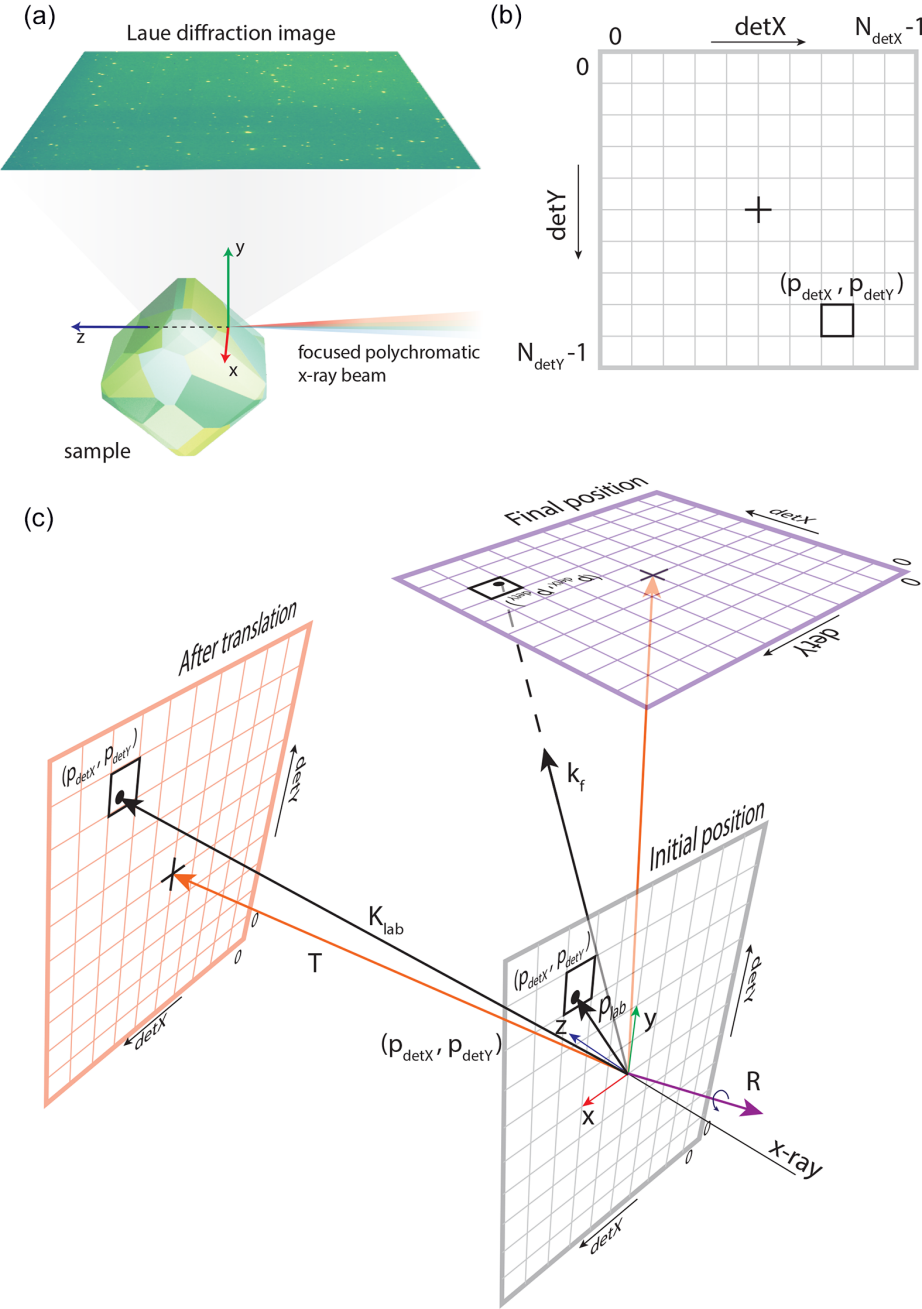
(*a*) Schematic drawing of the Laue diffraction experiment. The focused polychromatic beam is incident on the sample from the right. The detector is placed at 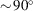 from the X-ray beam propagation direction. (*b*) Transforms to take pixel position on the detector in (*a*) to the detector coordinate system. (*c*) Detector in the laboratory coordinate system defined with vectors 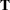 and 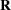: initial position (gray) before transformation, position after translation by 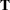 (orange) and final position after rotation by 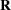 (purple).

Fig. 1 ▸ shows a schematic view of the Laue experiment in the reflection geometry at the Laue microdiffraction station 34-ID-E at the Advanced Photon Source. Such a configuration is common for Laue instruments at other synchrotrons. A polychromatic X-ray beam (spectrum of photon energies up to 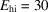 keV) from an undulator source is incident on a poly- or single-crystalline sample. A Laue diffraction pattern is formed on the 2D detector, nominally positioned at a distance *L* above the sample at a scattering angle centered at 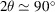 [Fig. 1 ▸(*a*)]. The detector frame size is 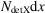 by 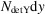, where 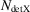, 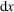 and 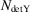, 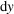 are the number of pixels and the pixel size along the first (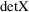) and second (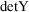) dimensions, respectively, shown in Fig. 1 ▸(*b*). The detector position and orientation in the laboratory frame (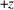 is the X-ray beam direction, 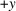 is up and 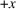 is outboard of the synchrotron ring) can be defined by two vectors: a translation vector 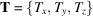 and a rotation vector 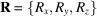.

To define the detector position using 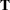 and 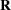, we start with the center of the detector matrix at 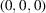 of the laboratory coordinate system; the detector matrix is normal to the incident beam and is oriented with its first dimension (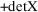) along 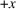; the second dimension (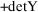) is along 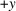. This is denoted as the detector coordinate system, shown schematically in Fig. 1 ▸(*c*) (initial position). The detector is first translated by the vector 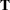 [Fig. 1 ▸(*c*) (after translation)] and then rotated about the vector 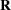 to arrive at the final position in the instrument [Fig. 1 ▸(*c*) (final position)]. In our notation, the magnitude of the vector 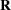 is the angle (in radians) by which the detector is rotated. Before an experiment, the 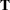 and 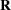 vectors are calibrated using a Laue diffraction pattern from a thin single-crystal Si sample with a known orientation positioned at the origin of the coordinate system.

Forward simulation, that is, computing the location of diffraction signal on the detector from an oriented crystal, involves these steps in reverse for each allowed *hkl*. The diffraction vector, according to the orientation of the crystal, is first computed in the laboratory coordinate system. This position is then rotated by 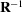 and un-displaced by 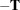 to determine the pixel position on the un-displaced initial detector position lying in the *XY* plane. The procedure is as follows below.

We define initial crystal orientation using the orientation matrix, *U*, which transforms a vector from the crystal frame to the laboratory frame. The rotated reciprocal-space matrix, 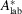, is computed as 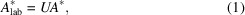
where 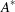 is the reciprocal-space matrix calculated from the lattice parameter of the crystal. This, combined with a valid vector of {*hkl*} indices for the crystal structure, yields 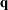 as 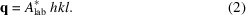
The unit vector in the direction of the diffraction signal, 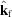 [shown in Fig. 1 ▸(*c*)], is 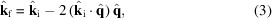
where 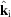 is the unit vector in the incident-beam direction and 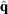 is the unit vector in the direction of the diffraction-plane normal, computed as 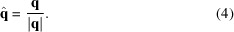
We now compute a rotation matrix to rotate from the detector frame to the laboratory coordinate system using 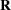: 
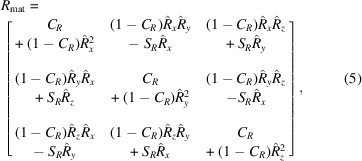
where 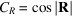, 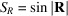, 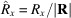, 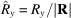 and 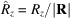. This is the standard matrix formulation for an axis–angle rotation, where the vector 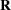 defines the axis of rotation and its magnitude, 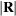, defines the angle of rotation in radians.

The inverse of 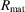 can be used to rotate 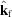 to the detector coordinate system (before application of 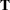) as
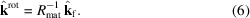
The term 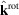 is then scaled to the *z* displacement of the detector to compute 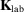 as 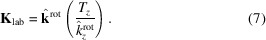
Here 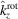 is the component of 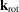 along the *z* axis in the initial (unrotated, undisplaced) detector coordinate system, representing the projection onto the detector-plane normal.

The position of the diffraction signal on the detector in the laboratory coordinate system after displacement of 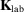 by 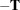 is
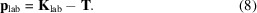
As described earlier, at this step, the middle of the detector is located at the origin of the laboratory coordinate system [Fig. 1 ▸(*c*)]. Thus, the position of the diffraction signal on the detector (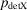 and 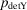 element indices in the 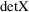 and 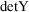 directions, respectively) is computed from 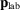 by first transforming to pixel coordinates by dividing by the respective pixel size and then displacing the origin to the edge of the detector [Fig. 1 ▸(*b*)] as follows: 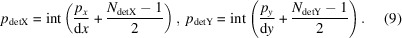
The X-ray energy of the corresponding signal is 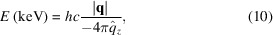
where 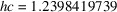
 keV nm. The following filters are subsequently used to determine valid diffraction signal: 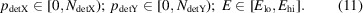


## 3. Workflow

Before detailing the workflow, we will define several key terms to ensure clarity. A crystal orientation refers to the rotational transformation of a grain’s crystallographic axes relative to the laboratory coordinate system. The terms ‘strong orientation candidate’ or ‘weak orientation candidate’ do not describe intrinsic properties of the orientation itself but rather the quality of the match between the simulated pattern for that orientation and the experimental data. A strong orientation candidate corresponds to a high figure of merit, indicating a significant diffraction signal, likely from a large or well oriented grain. Conversely, a weak orientation yields a lower figure of merit and typically corresponds to a weaker diffraction signal.

The *LaueMatching* workflow, detailed below, is structured into three key stages designed to efficiently generate the necessary simulated templates, prepare the experimental data for robust matching and perform the final orientation determination:

(i) Generation of the library of diffraction signals.

(ii) Pattern preprocessing.

(iii) Indexing and refinement of orientations and crystal structure.

The following sections describe these steps in more detail.

### 3.1. Generation of the library of diffraction signals

**Figure 2:**
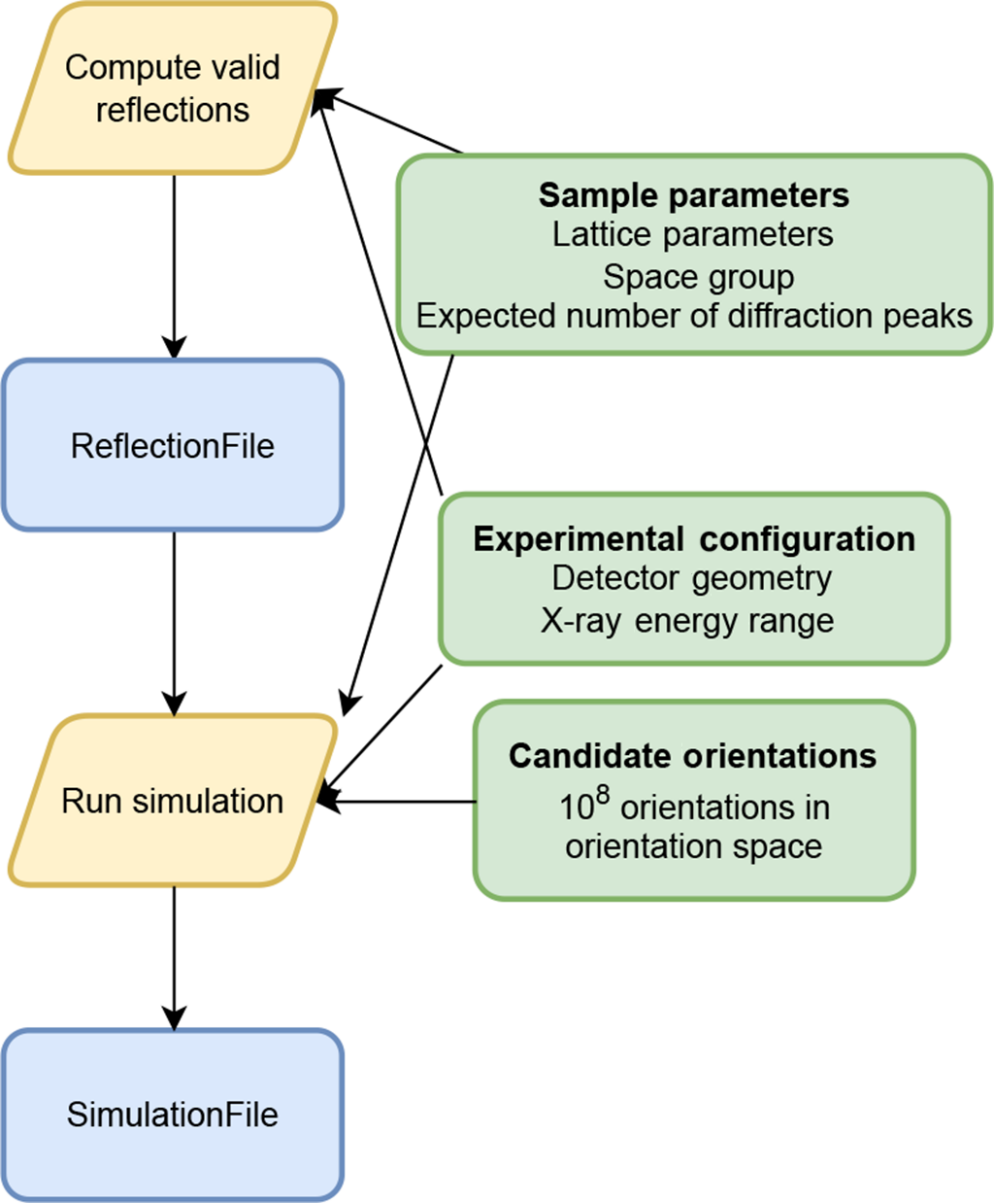
Schematic diagram of workflow to generate the *SimulationFile* in *LaueMatching*. Blue boxes indicate file I/O to the system, green boxes indicate user inputs and yellow boxes indicate computation.

In this step, the forward-simulation model generates a comprehensive library of *hkl* detector positions for a certain experimental configuration, crystal structure and orientation grid. If the material consists of multiple phases, this procedure is repeated separately for each phase, and the workflow is applied independently each time. Fig. 2 ▸ shows the workflow to generate the library of signals. The required input is divided into three categories:

(i) Sample parameters. These correspond to the phase of interest. The parameters supplied are the space group, crystal lattice parameters and expected number of diffraction peaks (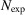). The expected number of diffraction peaks is the number of peaks resulting in signal on the detector in the current experiment configuration. 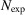 serves as an estimate to optimize the size of the simulation library and does not need to be exact. It is used to pre-allocate memory and limit the number of reflections stored for each orientation, with a reasonable value easily estimated from a single forward simulation.

(ii) Experimental configuration. This corresponds to the experimental setup. The parameters supplied are detector position (experimentally calibrated translational and rotational vectors 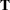 and 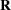; see Section 2 for more details), pixel size (d*x*, d*y*) and number of pixels of the detector (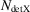, 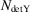), and range of X-ray energies used to filter allowed diffraction peaks in the simulation (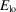, 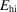).

(iii) Candidate orientations. This is a list of orientations (supplied as quaternions and saved in an *OrientationFile*) that will be used for simulation. Typically, this list includes uniformly spaced orientations on a regular grid in the full orientation space, not just in the fundamental zone for the corresponding crystal structure. This is deliberate, as using the full space allows for a robustness check during indexing by requiring multiple symmetry-equivalent orientations to be found (see Section 3.3). The orientation grid can be generated using programs based on *EMsoft* (Singh *et al.*, 2017 ▸), *Orix* (Johnstone *et al.*, 2020 ▸), *Neper* (Quey *et al.*, 2018 ▸) or *MTEX* (Krakow *et al.*, 2017 ▸). For example, 0.6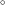, 1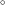 and 2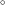 orientation spacing results in ∼100 million, 20 million and 2.4 million orientations in the full orientation space, respectively. The *LaueMatching* repository includes an example file and example codes for the generation of the *OrientationFile*.

The first stage in this workflow involves the computation of a set of valid reflections, corresponding to [*hkl*] directions, that are allowed for diffraction after computing the lattice extinction rules for the assumed crystal structure. Reflections prohibited due to the structure factor (basis forbidden *hkl*s) are not excluded at this stage. This list of *hkl*s is saved to a *ReflectionFile*.

The next step is to generate the simulation. For each orientation in the candidate orientation list, a sub-set of *hkl*s is computed that satisfies the following conditions: the diffraction signal lands on the calibrated detector position and falls within the specified X-ray energy range. The positions of up to 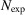 valid diffraction peaks (sorted by *d* spacing) are saved in a *SimulationFile*. These positions are saved as pixel coordinates on the detector; doing this significantly reduces (i) the data size by reducing the highest stored values {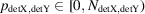} from equation (11) and (ii) the computation time during indexing by efficient indexing, described more in Section 3.3.

*OrientationFile*, *ReflectionFile* and *SimulationFile* are gen­erated once for each crystal structure and experimental configuration. This orientation library is reused for any experimental diffraction analysis where the sample satisfies the crystallographic parameters. New libraries of patterns can be pre-computed for new crystal structures as needed. The simulation library is specific to a given experimental geometry. If the detector position or other geometric parameters change, a new library must be generated. It is important to note the favorable scaling: the computationally intensive library generation is a one-time cost per configuration, while the indexing of individual patterns is extremely rapid, making the approach highly efficient for large datasets.

### 3.2. Pattern preprocessing

One unique feature of *LaueMatching* is that fitting peaks to obtain peak positions is unnecessary; the algorithm computes a correlation using the raw diffracted signal between the simulated diffraction peak positions and the corresponding intensity on the detector. This direct use of intensity avoids the challenge associated with precise peak finding and fitting, particularly for weak, overlapping or distorted peaks, which can affect traditional methods. The indexing process, described in the next section, selects a smaller set of orientations from the millions of candidate orientations computed in the library of diffraction signals. These are then refined to get the orientations present in the sample.

Computing the correlation between the experimental data and the pre-computed diffraction signal for an orientation is significantly faster than the refinement step. Thus, reducing the number of orientations that need to be refined is computationally advantageous. This is achieved by pre-processing the diffraction patterns to isolate the diffraction signal from the background. By applying a threshold after background subtraction, pixels not containing diffraction information are set to zero counts. This is distinct from traditional peak finding, as it does not involve fitting peak profiles or finding peak centers, but rather prepares the entire diffraction signal for correlation. This threshold is applied globally on a per-frame basis.

The strict computation of the correlation function and a finite spacing of orientations necessitates incorporating the difference in the real orientation and its closest neighbor in the chosen orientation grid. (The maximum difference in these can be 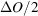, where 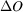 is the orientation-grid spacing.) Seret *et al.* (2022 ▸) used a similar approach with a regularly spaced orientation grid but applied an angular uncertainty to the experimental plane normal vectors each time when matching with the simulation. This approach is computationally expensive for the millions of orientations tested in *LaueMatching*. Thus *LaueMatching* uses a reverse approach: the thresholded experimental diffraction pattern is blurred with a Gaussian filter of width *W*, computed as follows: 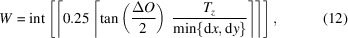
where 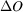 is the orientation spacing in radians and 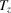 is the sample-to-detector distance, assuming nominal 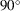 geometry. This assumes that the blurring effect extends to 4*W* pixels.

**Figure 3:**
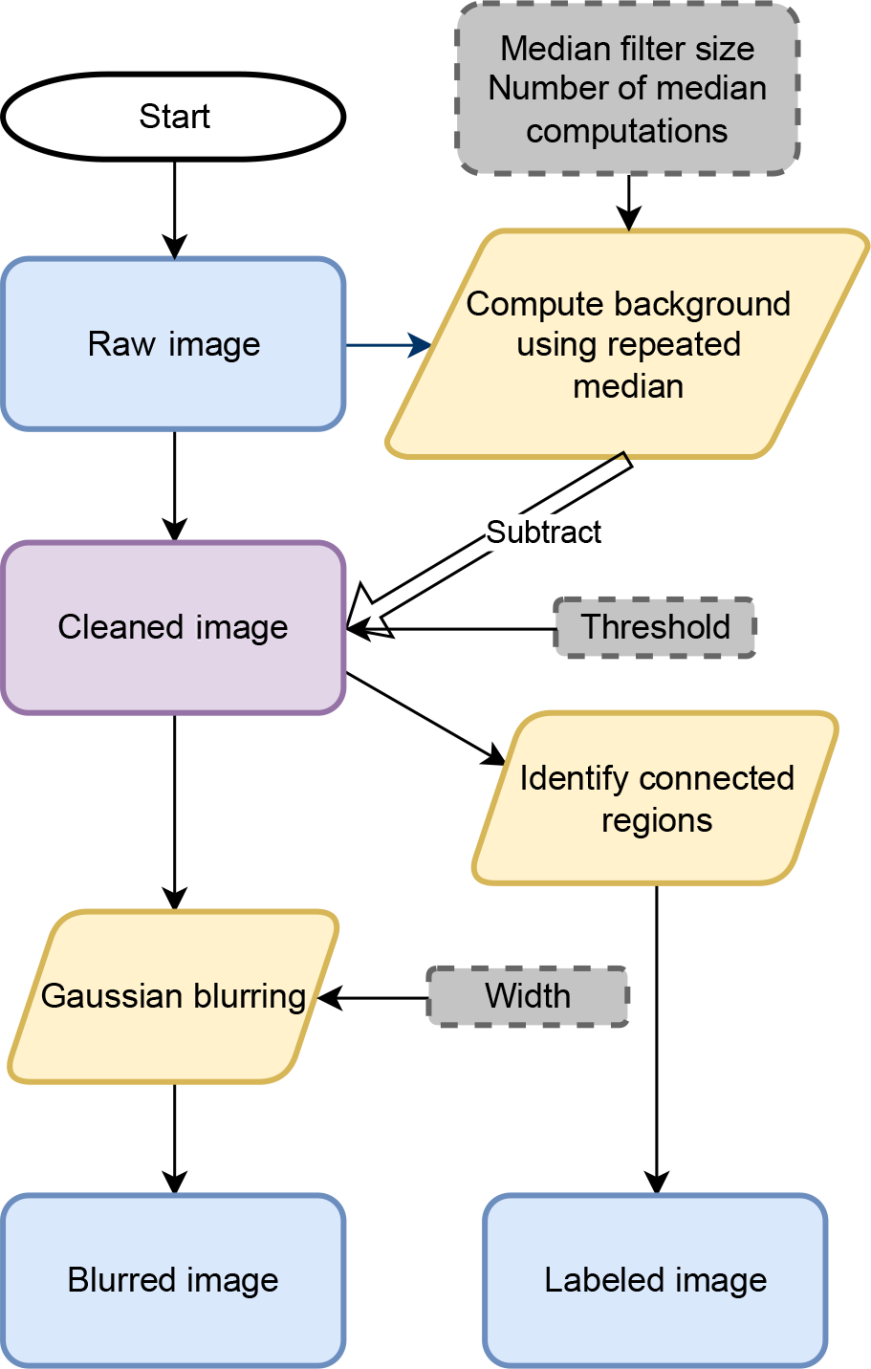
Schematic diagram of the pattern-processing workflow in *LaueMatching*. Blue boxes indicate file I/O to the system, yellow boxes indicate computation, gray boxes with dashed border indicate optional user inputs and purple boxes indicate intermediate stored files.

**Figure 4:**
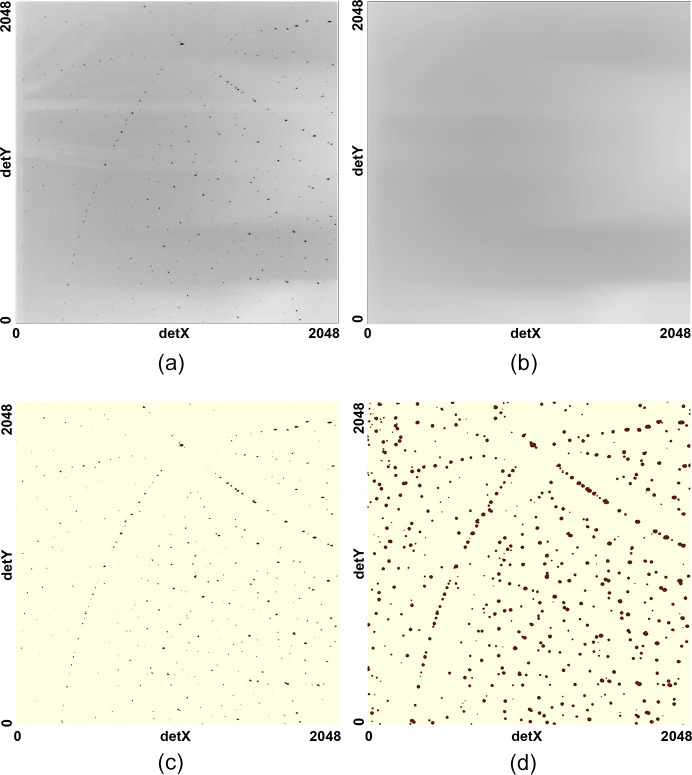
Pattern-processing results for the 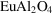 example. (*a*) Raw pattern. (*b*) Computed background using the repeated median. (*c*) Cleaned pattern. (*d*) Gaussian blurred pattern. No manual inputs were provided, and the procedure was run automatically to compute the inputs at each step in Fig. 3 ▸.

The pattern-processing workflow is shown in Fig. 3 ▸, and Fig. 4 ▸ shows the results for an example diffraction pattern.

The pattern-processing workflow consists of the following steps:

(1) The first step involves computing the background signal on the image. This is done by repeated application (4–5 times) of a median filter on the image. The median-filter size is ∼4× the size of the largest diffraction peak on the image using the *DIPlib* library (*DIPlib*, 2025 ▸). Fig. 4 ▸(*b*) shows the computed background for the diffraction image in Fig. 4 ▸(*a*). This background is subtracted from the image. The empirically determined background may be used on different images acquired using the same experiment configuration.

(2) The next step involves the computation of a threshold to be applied to the background-corrected image. This threshold is typically 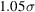 of the background-corrected image or the intensity of 90th percentile of the pixels, where σ is the standard deviation. All pixels with intensity below this threshold are set to 0.

(3) A connected-components operation on the cleaned image is used to determine connected regions of the image. These connected components are used later to identify unindexed regions in the pattern for subsequent reprocessing.

(4) The Gaussian blurring filter of width σ is applied to the image. The image datatype is converted to ‘double’ to preserve fractional intensities.

The Gaussian blurring step is critical for the algorithm’s robustness. It effectively ‘smears’ the intensity from each pixel, allowing a successful match to be found even if the true grain orientation lies between the discrete points of the simulation or if minor experimental misalignments cause small shifts in peak positions. It is this feature that allows *LaueMatching* to achieve high-precision orientation refinement even with deliberately introduced peak-position noise, as quantitatively benchmarked in our simulated ground-truth analysis in Section 4.

### 3.3. Indexing and subsequent refinement

**Figure 5:**
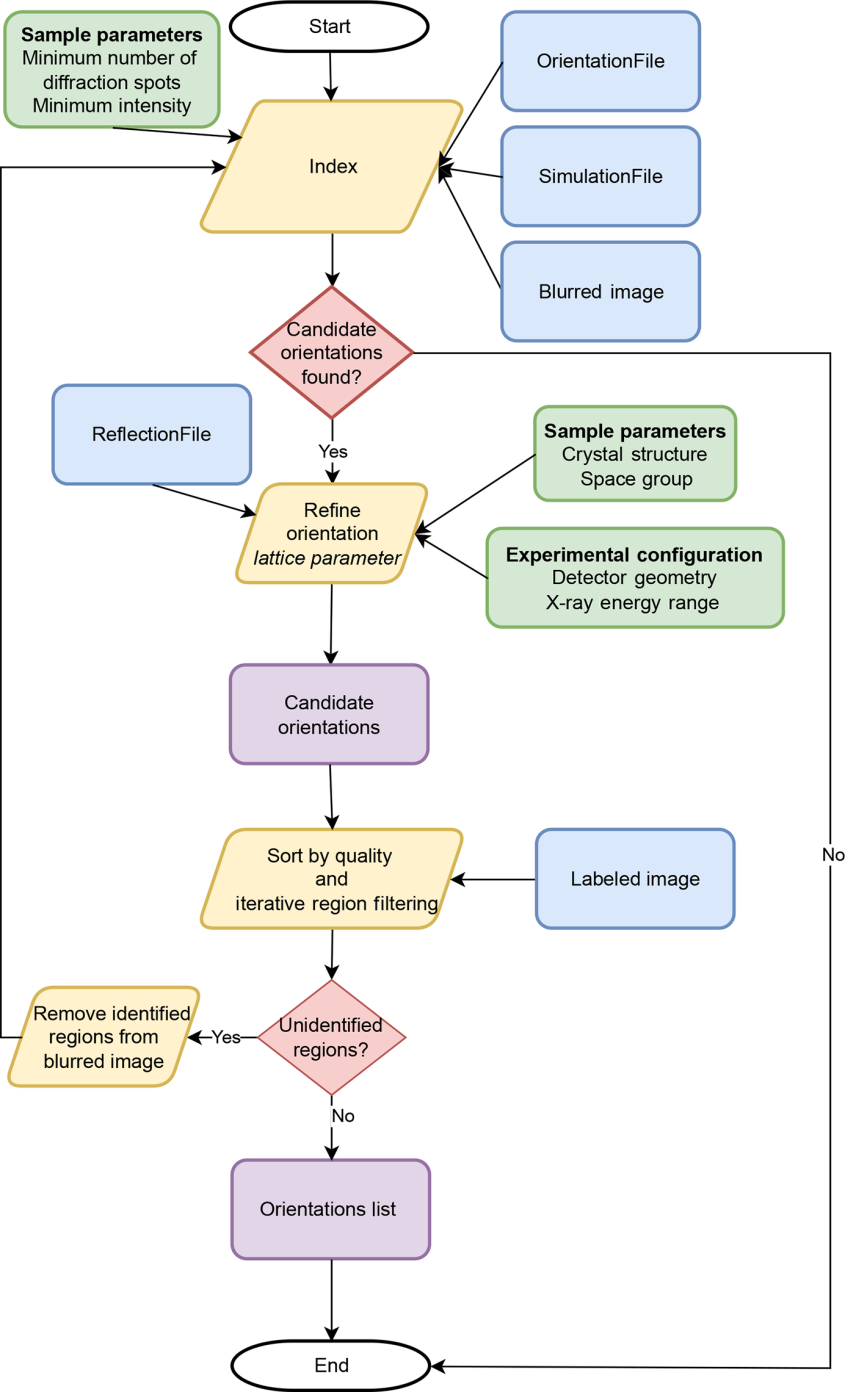
Schematic diagram of the indexing and refinement workflow in *LaueMatching*. Blue boxes indicate file I/O to the system, green boxes indicate user inputs, yellow boxes indicate computation, red boxes indicate decision steps and purple boxes indicate intermediate stored files. The computation can be run on GPU or CPU resources.

Fig. 5 ▸ describes the workflow for determining orientations from a pre-processed pattern. This can be split up into two main parts: indexing and refinement. In the indexing step, diffraction peaks are assigned to different orientations, and the orientation and lattice parameters are refined in the refinement step.

The first step is to run a look-up table search. For each orientation in the *OrientationFile* and each corresponding pixel position of the simulated peaks in the *SimulationFile*, experimental signal intensity is obtained by accessing the corresponding position in the diffraction pattern. The intensity sum 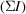 is computed by adding the intensity values for all the peak pixel positions in the *SimulationFile* for the corresponding orientation. The term 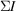 and the number of peak positions with non-zero intensity (*N*) (pattern preprocessing ensures that only pixels belonging to the diffraction signal have non-zero intensity) are used to define the figure of merit (*F*) in different user-selectable ways: 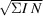, 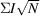, 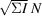, 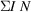 and 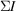.

These options allow users to tailor the search sensitivity according to the experimental conditions; for instance, weighting *N* higher might be beneficial when many peaks are expected but they are potentially weak, while weighting 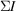 higher prioritizes stronger, potentially fewer, signals. While several figures of merit are available to tailor the search sensitivity, we found 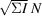 to be the most robust and reliable metric across a wide range of experimental conditions. It effectively balances the need to find patterns with a sufficient number of peaks (*N*) and strong total intensity 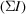. We therefore recommend it as the default for most applications. An orientation candidate is considered for subsequent refinement if the figure of merit exceeds 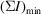 and 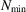. The term 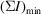 is detector-signal and crystal-quality dependent; typically, a value of ∼100 counts is used to detect weak signal. The term 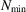 depends on the detector configuration and the material’s crystal structure. A good rule of thumb is to use 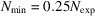, where 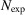 is the number of expected diffraction peaks from a random orientation resulting in signal on the detector. This 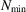 value accounts for missing reflections due to non-uniform X-ray beam energy profiles, weakly reflecting materials and structure-factor extinctions. Optimal values for these thresholds may require minor tuning depending on the specific material system and experimental signal-to-noise levels, though the provided rule of thumb for 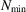 offers a good starting point.

The list of candidate orientations found is then further processed. If multiple orientations are detected, the algorithm can optionally operate iteratively: identified peaks corresponding to strong orientation candidates are masked (conceptually removed) from the blurred pattern and the search process (look-up table search and *F* calculation) is repeated on the residual pattern. This helps in identifying weaker orientation candidates obscured by stronger ones. The connected components identified during preprocessing [Section 3.2, step (3)] can be used to guide this masking or analyze remaining unidentified regions. The figure-of-merit approach, summing intensity over expected peak locations even if some are missing or weak, inherently handles cases with low numbers of detected peaks or missing reflections, a challenge for methods relying on inter-peak angles. This iterative masking procedure is particularly crucial for disentangling heavily overlapped signals, as will be demonstrated in the successful indexing of 28 overlapping orientations in the complex polycrystalline Al-wire example in Section 4 (Fig. 8).

Using the full orientation space instead of only the fundamental zone of the crystal structure for simulation enables *LaueMatching* to apply another filter to the solutions; each unique orientation must be matched multiple times and orientations below this criterion are filtered out. This increases the execution time for searching by up to 24 times (for cubic crystal systems, the fundamental zone is 24 times smaller than the full orientation space) and adds duplicate solutions, but it has been observed to be very robust by removing low-quality or spurious matches. This redundancy check enhances the robustness compared with methods that might identify a single, potentially spurious, match. This is a deliberate methodological choice. While the space group is used initially to determine the list of allowed reflections, we do not restrict the search to the fundamental zone. Instead, searching the full orientation space allows us to use the discovery of multiple symmetry-equivalent orientations as a powerful filter for validation, which significantly enhances the robustness of the final result by eliminating spurious matches.

The refinement process optimizes the orientation parameters and, optionally, selects lattice parameters by maximizing the figure of merit (*F*) using the *NLopt* non-linear optimization library (Johnson, 2025 ▸). The optimal parameters are determined automatically by the optimization algorithm, which iteratively seeks the values that maximize the figure of merit. In a white-beam Laue experiment, a change in a lattice parameter is indistinguishable from a scaling of the incident photon energies. Because the energy for each reflection is not measured, this ambiguity prevents a full unconstrained refinement of the unit-cell volume. Therefore, *LaueMatching* refines lattice parameters under a constraint of constant unit-cell volume (*e.g.* by only optimizing the *c*/*a* ratio for hexagonal close-packed structures). The overall experimental geometry (described by vectors 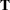 and 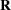) is considered fixed during indexing, having been determined in a prior calibration step. The number of diffraction peaks for matching is increased up to 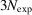 during refinement to detect weaker diffraction peaks.

## 4. Experimental verification

Laue datasets from Ni, Al and 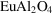 samples were collected at the Advanced Photon Source using the Laue microdiffraction station 34-ID-E. A polychromatic X-ray beam (7–30 keV) from an undulator source is incident on a polycrystalline sample. The simulation range (6–30 keV, see Section 2) was chosen to be slightly wider to ensure capture of all potential peaks near the experimental energy bandpass. A Laue diffraction pattern is formed on the 2D detector (PerkinElmer, 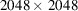 matrix with square pixels of 200 µm), positioned ∼500 mm above the sample at a scattering angle centered near 2θ 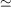 90°. Experiment parameters and lattice parameters for each sample are given in Table 1 ▸. *LaueMatching* was run with a candidate orientation list with 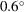 orientation spacing, consisting of 100 million orientations. Lattice parameter refinement was switched off for all datasets except for the 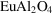 sample.

**Figure 6:**
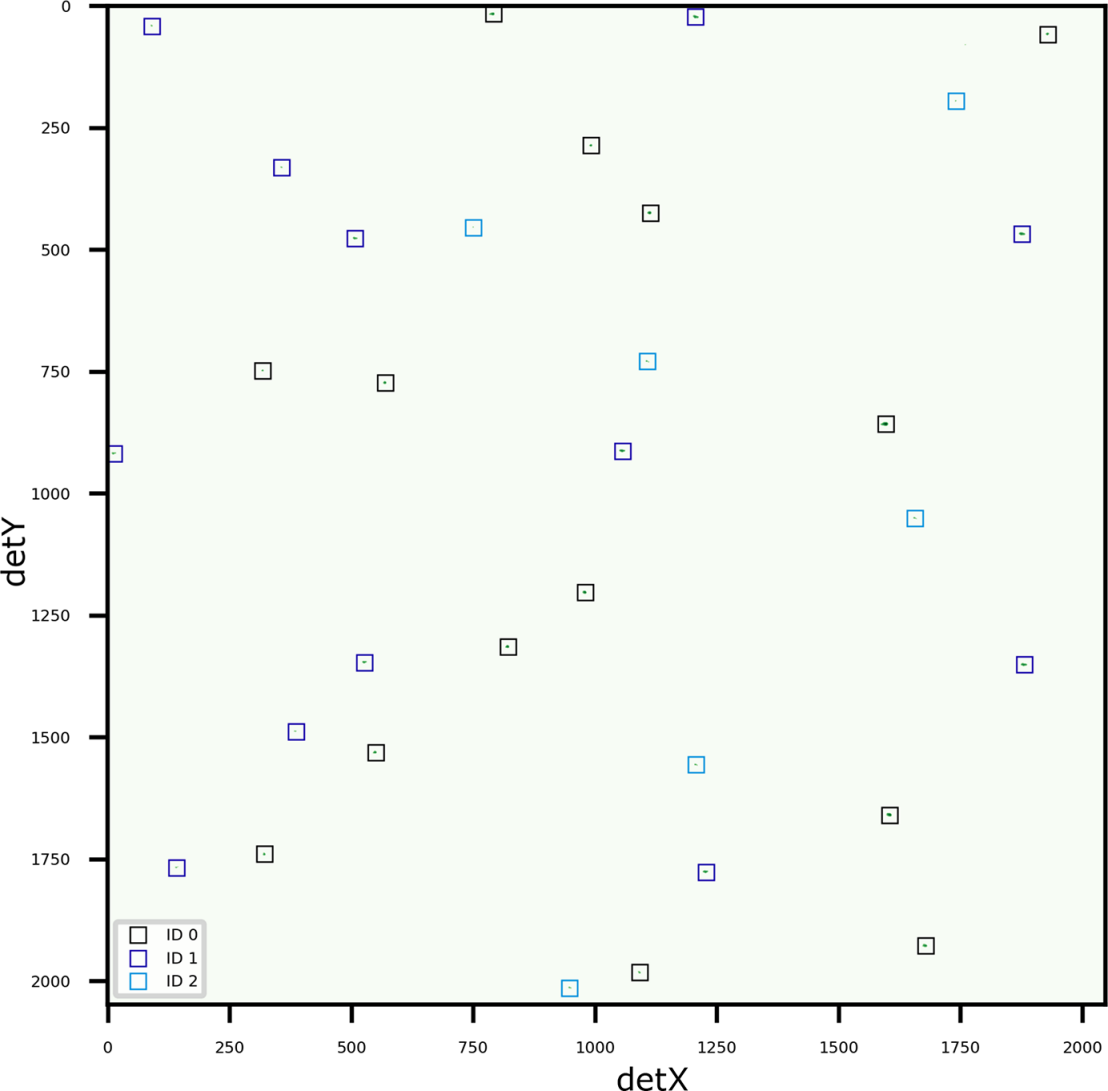
Diffraction pattern of an Ni sample with three indexed orientations. The fitted peak positions are overlaid as boxes colored according to refined orientations from *LaueMatching* on the cleaned diffraction pattern. Note the lack of any obvious zones for any orientation.

The first example of an Ni sample with three grain orientations, acquired without depth resolution (Gürsoy *et al.*, 2022 ▸), is shown in Fig. 6 ▸. All three orientations were identified using *LaueGo* (Tischler, 2014 ▸). *LaueMatching* was able to identify the same three weak-signal orientations with only 70%, 60% and 30% of expected peaks detected on the pattern, with 14, 12 and 7 peaks identified for each corresponding orientation, respectively. The misorientation angles between the solutions from the two methods (*LaueMatching* and *LaueGo*) were exceptionally low – 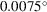, 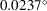 and 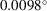, respectively – demonstrating excellent agreement.

**Figure 7:**
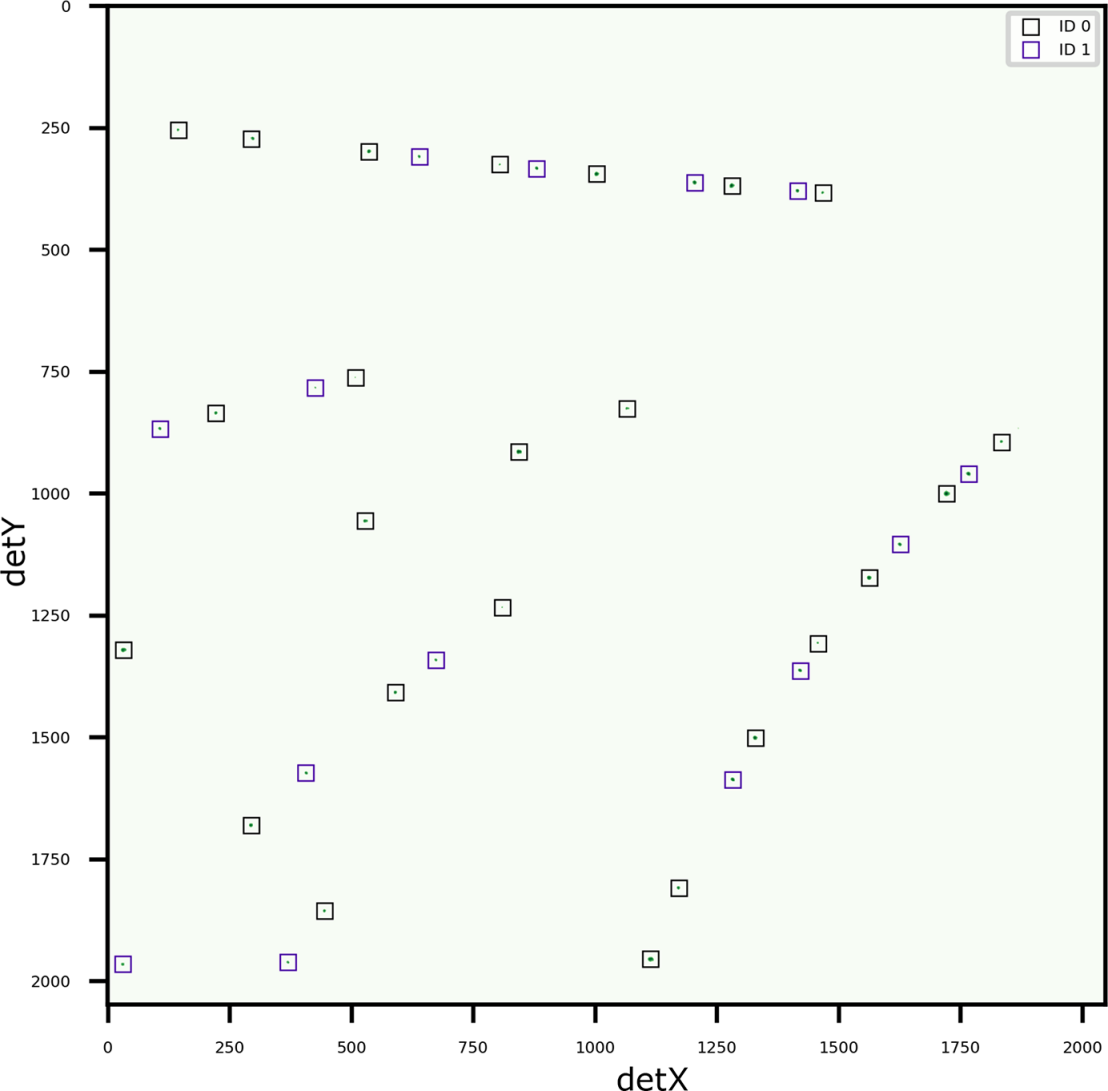
Diffraction pattern of an Ni sample with two indexed orientations, where one is the crystallographic twin of the other. The fitted peak positions are overlaid as boxes colored according to refined orientations from *LaueMatching* on the cleaned diffraction pattern.

The second example consists of an 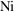 sample with two grain orientations, where one orientation is a crystallographic twin of the other; this specifically tests the handling of twins, which present a unique challenge because some *hkl*s are shared between the two orientations (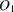 and 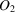) (Fig. 7 ▸). The ideal misorientation between crystallographic twins is 60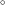 around 〈111〉 for face-centred cubic structures. Three of the shared *hkl*s, 753, 846 and 113 (113 in 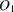 is the same as 717 in 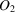), could be resolved by using an advanced pattern-processing technique such as watershed (Sharma *et al.*, 2012 ▸). However, 135, 246 and 133 (133 in 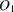 is the same as 11 5 5 in 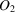) are indistinguishable even using watershed. *LaueMatching* suc­cess­fully identified the two orientations and assigned the correct indices to each peak. This demonstrates the robustness of *LaueMatching* using the correlative approach. *LaueMatching* successfully assigned the shared *hkl*s to the two orientations. The misorientation between 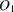 and 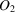 was 59.978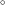 around the [111] direction. Overall, 24 peaks were assigned to 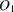 and 21 to 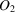.

**Figure 8:**
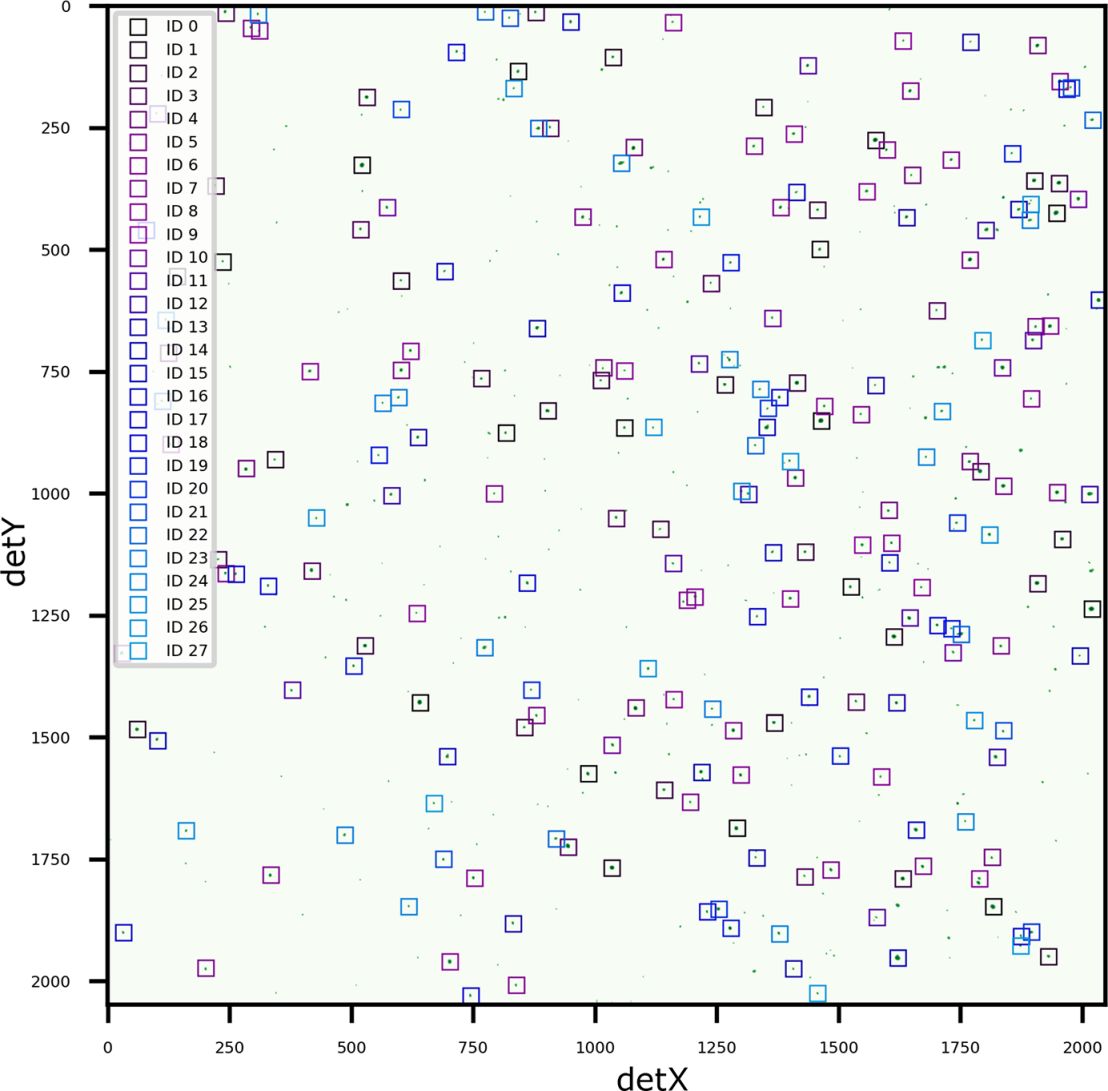
Diffraction pattern of an Al sample acquired without depth resolution, resulting in heavily overlapped signals from multiple grains. The fitted peak positions for 28 distinct orientations identified by *LaueMatching* are overlaid as colored boxes, demonstrating the algorithm’s ability to index high-multiplicity patterns common in polycrystalline samples.

In the third example, a Laue diffraction pattern was acquired on an 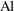 wire without depth resolution; the diffraction signal from multiple grains superimposed significantly, creating a complex pattern typical of polycrystalline aggregates measured without depth resolution, as shown in Fig. 8 ▸. *LaueMatching* found 28 separate grain orientations in the pattern, illustrating the ability to resolve a high number of overlapping orientations within a single pattern. The validity of these 28 orientations was confirmed by comparing them with results obtained using *LaueGo* on corresponding depth-resolved data (acquired up to 300 µm depth), with all orientations matching within a 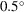 misorientation tolerance. Successfully resolving such a high density of orientations, ranging from strongly diffracting grains to weaker ones (indicated by the range of 5–19 peaks assigned to grains), highlights the effectiveness of the correlation approach combined with the iterative masking procedure (described in Section 3) in disentangling heavily overlapped signals.

**Figure 9:**
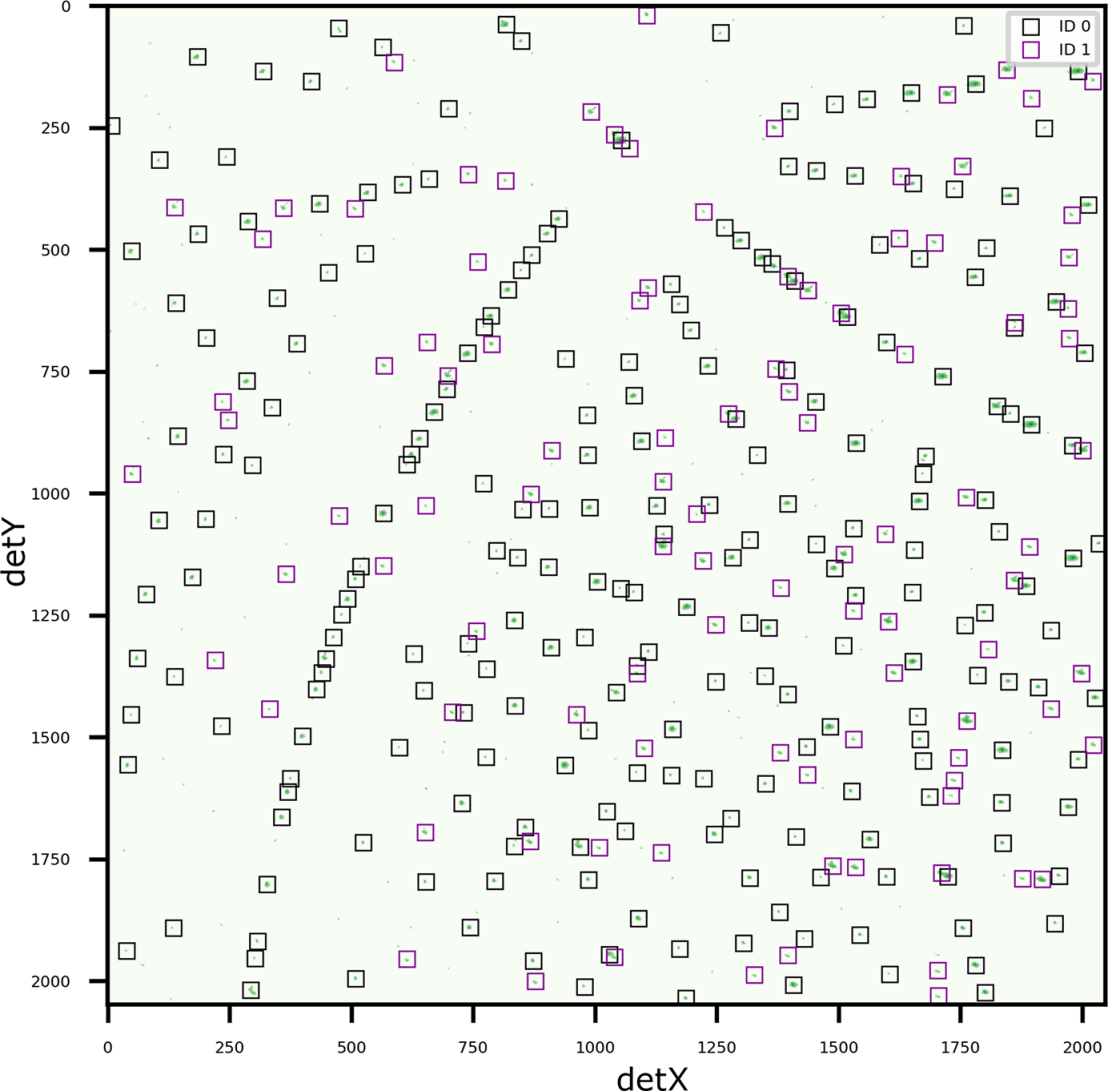
Diffraction pattern of an 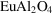 sample with two indexed orientations. The fitted peak positions are overlaid as boxes colored according to refined orientations from *LaueMatching* on the cleaned diffraction pattern.

Next, *LaueMatching* was used on a diffraction pattern collected using an 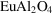 sample (Liu *et al.*, 2013 ▸), demonstrating indexing of a more complex (orthorhombic, space group 4) crystal structure and the optional lattice parameter refinement capability. Fig. 9 ▸ shows the diffraction pattern. *LaueMatching* was run using an intentionally deviated lattice parameter, shown in Table 1 ▸, up to 5% from the reference value in the literature (Tezuka *et al.*, 2013 ▸), and the unit-cell volume was conserved. *LaueMatching* was able to detect two orientations, separated by 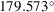. The successful indexing of this complex orthorhombic structure, even starting from intentionally deviated lattice parameters, highlights the effectiveness of the optional constant-volume lattice refinement capability described in Section 3.3. The two orientations were assigned 206 and 81 peaks. The term 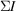 for the two orientations was 57 735 and 16 888, respectively, indicating that the second orientation had a much weaker signal. Furthermore, Table 1 ▸ shows that the refined lattice parameter was within 0.5% of the reference value (Tezuka *et al.*, 2013 ▸). The details of lattice parameter refinement are given in Section 3.3.

**Figure 10:**
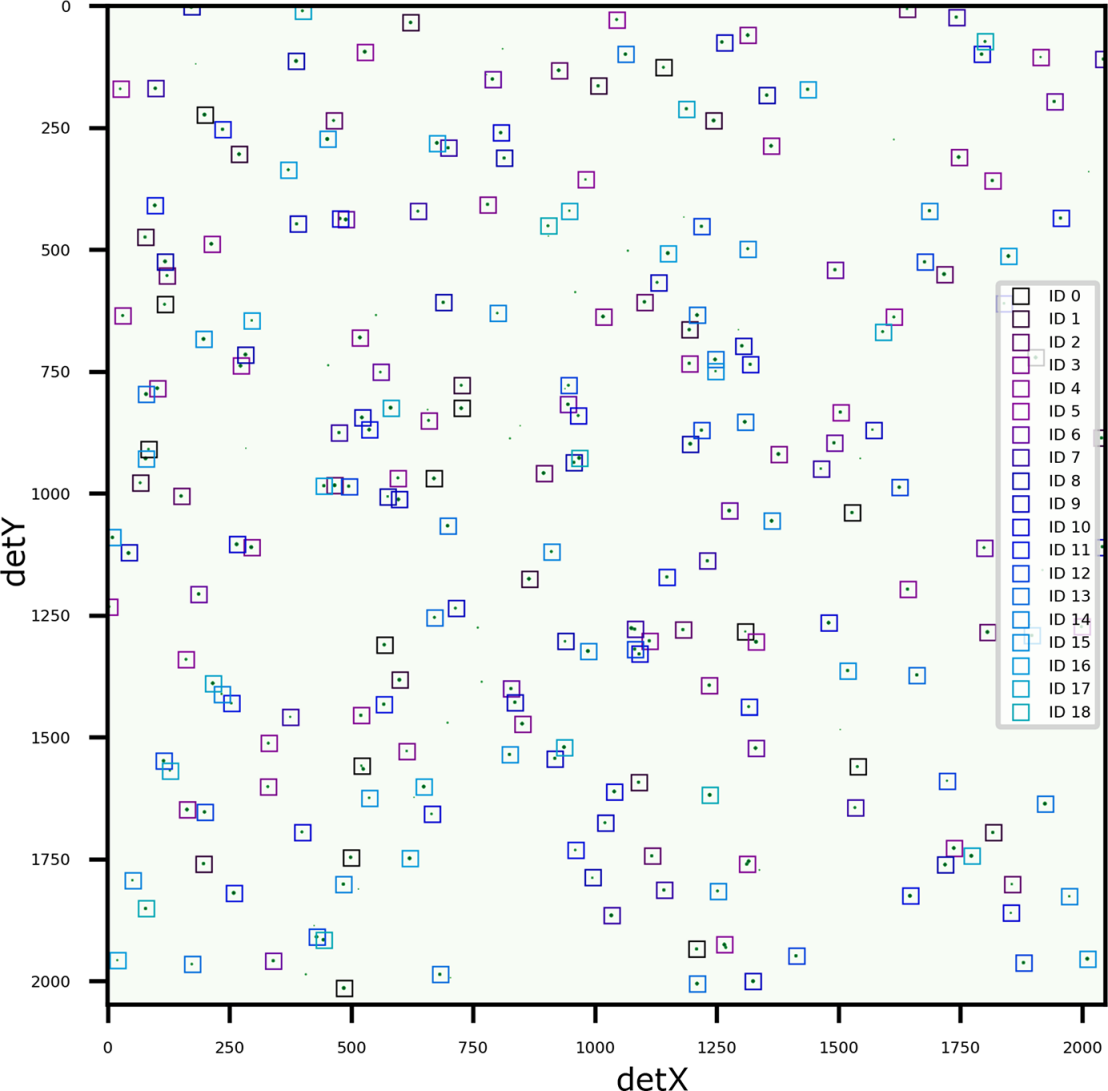
Simulated diffraction pattern of an Ni sample with 19 simulated orientations. All 19 orientations were successfully indexed. The fitted peak positions are overlaid as boxes colored according to refined orientations from *LaueMatching* on the simulated diffraction pattern. The simulation and reconstruction parameters are given in Table 1 ▸.

**Figure 11:**
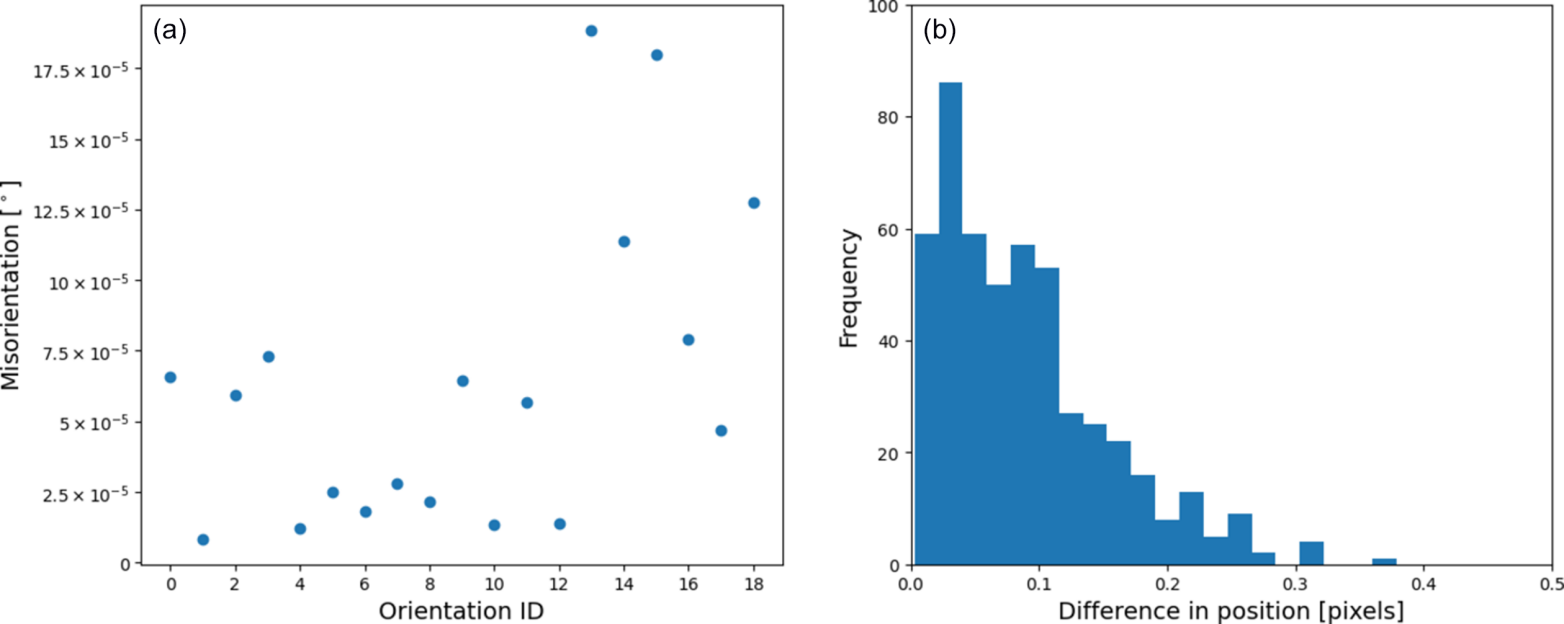
For the simulation shown in Fig. 10 ▸, (*a*) gives the misorientation, in degrees, between the orientations used to simulate the diffraction pattern (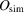) and the refined orientations from *LaueMatching* (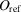). (*b*) A histogram plot of Euclidean distance between the computed peak positions from 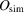 and 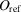. The mean difference is 0.088 pixels, while the maximum is 0.379 pixels.

Finally, to provide a quantitative benchmark against a known ground truth, a simulated diffraction pattern was generated using the same experiment configuration and crystal system as the Ni samples with 19 randomly chosen orientations. The simulation involved computing diffraction peak positions on the detector with an energy range of 6–30 keV and randomly discarding between 30 and 60% of the peaks. The positions of the remaining peaks were randomly perturbed by up to half a pixel, and Gaussian peaks were added to the diffraction pattern at each peak position with random heights up to 16 384 counts, covering the full 14-bit dynamic range of a simulated detector. *LaueMatching* was run to detect orientations with a minimum of 30% of expected peaks. As shown in Fig. 10 ▸, all 19 orientations were detected, with 6–15 peaks assigned to each orientation. Fig. 11 ▸(*a*) shows the misorientation angle between the input and refined orientations. All angles are in degrees. The resulting misorientation angles were exceptionally low, with all 19 orientations matching the ground truth to within 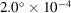, highlighting the high precision of the refinement algorithm. The mean misorientation angle is 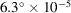, while the minimum misorientation angle is 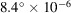. Such low misorientation angles highlight the strength of *LaueMatching*, which can robustly refine orientations without requiring the precise fitting and centroiding of individual diffraction peaks. Fig. 11 ▸(*b*) shows the difference in the computed position of diffraction peaks according to input and refined orientations. Even though the diffraction peaks were randomly displaced by up to half a pixel before running *LaueMatching*, the orientations detected using *LaueMatching* result in a computed diffraction signal located within 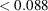 pixels, on average, from the diffraction signal computed from the input orientations. Achieving such high refinement accuracy, even with deliberately introduced peak-position noise (up to half a pixel) and missing peaks in the input simulation, underscores the algorithm’s resilience to typical experimental imperfections and its ability to converge correctly without precise initial peak fitting.

In each of the examples above, *LaueMatching* was run on two different systems: one workstation with 64 CPU cores of AMD EPYC 9174F CPUs and another workstation with a NVIDIA H100 GPU with 80 GB RAM. The single-pattern runtime of *LaueMatching* was similar on both systems. The Ni and Al examples (cubic structure, 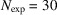) took ∼0.2 s to index, and the 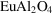 example (orthorhombic structure, 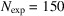) took ∼1 s to run. This base time scales with 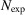, which determines the size and complexity of the *SimulationFile*. To approximate performance on more common hardware, *LaueMatching* was run on the AMD EPYC 9174F workstation utilizing only four of its 64 CPU cores for the cubic test cases. Indexing took 3.77 s in this scenario, a time comparable to execution on typical workstations or laptops.

The size of the *SimulationFile* is critical for determining parallel processing capabilities on memory-limited hardware like GPUs. For the cubic systems (Ni, Al), the *SimulationFile* was 12 GB, while for the more complex orthorhombic system (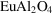), it was 72 GB (stored as uncompressed binary files). On the NVIDIA H100 GPU with 80 GB RAM, the available memory dictates how many instances can run concurrently per GPU.

Given that each Laue diffraction pattern can be indexed independently, significant throughput gains are possible on multi-GPU systems. For the cubic case, the 12 GB file size allows six instances of *LaueMatching* to be loaded concurrently on a single H100 GPU (6 processes 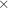 12 GB per process = 72 GB, fitting within the 80 GB RAM limit). Therefore on a supercomputer node like those on Polaris at the Argonne Leadership Computing Facility (https://www.alcf.anl.gov/polaris) equipped with eight such GPUs, a total of 48 processes (6 processes per GPU 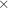 8 GPUs) can run in parallel. This configuration enables processing of up to ∼240 frames per second (FPS) for cubic structures.

However, for the more complex orthorhombic structure, the larger 72 GB *SimulationFile* only allows one instance per GPU to fit within the 80 GB RAM. Consequently, on an eight-GPU node, only eight processes can run concurrently. This results in a processing speed of up to 8 FPS for such complex crystal structures. These processing speeds facilitate near-real-time feedback during data acquisition or enable the efficient post-processing of large-scale microstructural maps containing thousands or millions of diffraction patterns.

Generation of the library of diffraction signals (*Simulation­File*) itself took 189 and 640 s for the cubic and orthorhombic crystal structures, respectively. Importantly, this *SimulationFile* needs to be generated only once for a given experiment configuration and crystal structure, representing a one-time computational cost amortized over potentially millions of analyzed patterns.

## 5. Limitations and future work

While *LaueMatching* demonstrates significant robustness, its performance relies on the quality and density of the pre-computed library. Generating dense libraries for very complex structures or extremely large orientation spaces can be computationally intensive, although this is a one-time cost per configuration. The effectiveness of the blurring and correlation approach may decrease in scenarios with extremely low signal-to-noise ratios where diffraction spots are barely distinguishable from background fluctuations after preprocessing. Similarly, the method’s performance relies on the ability to adequately subtract the background during preprocessing. In cases with highly non-uniform backgrounds, strong sample fluorescence or detector artefacts that are not well removed by the chosen background-estimation method, the remaining residual intensity could interfere with the correlation metric. That could potentially reduce sensitivity, particularly for weak signals, or impact refinement accuracy. To mitigate this, the preprocessing workflow in *LaueMatching* was specifically designed for robust background rejection. As demonstrated in the diverse experimental and simulated datasets, the combination of repeated median filtering and intensity thresholding proved highly effective for isolating the diffraction signal. The accuracy of lattice parameter refinement is limited by the energy bandwidth and geometric calibration precision inherent in Laue diffraction. Furthermore, while the correlation approach offers robustness, extreme inaccuracies in the initial geometric calibration could potentially impact orientation refinement accuracy, though less severely than methods reliant on precise peak metrology. Moreover, while the method is designed to be broadly applicable, the experimental validation in this work was performed on data from a single beamline; future work will involve testing *LaueMatching* on datasets from other facilities to confirm its robustness across different instrument geometries and sources. Future work could also explore adaptive grid refinement strategies for library generation, integrating more sophisticated noise modeling and investigating alternative figures of merit tailored for specific challenging conditions, such as extremely low signal-to-noise ratios or differentiating between near-degenerate orientations.

## 6. Summary

In summary, we have developed and introduced *LaueMatching*, a novel high-throughput indexing algorithm that robustly solves Laue diffraction patterns by directly correlating them against a pre-computed library of simulations. The method leverages a pre-computed library of simulated diffraction signals covering a dense sampling of orientation space. Experimental patterns are efficiently pre-processed to enhance diffraction features and suppress background before being directly correlated with the library entries using a figure of merit based on summed intensity at expected peak locations. This correlation approach intrinsically handles challenges like weak signals, peak overlap and missing reflections that hinder traditional peak-fitting methods. Subsequent refinement optimizes orientation and, optionally, lattice parameters. We demonstrated *LaueMatching*’s capabilities through validation on various experimental datasets, including multi-grain, twinned and complex crystal structures (Ni, Al, 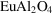), as well as on simulated patterns with known ground truth, consistently achieving rapid and highly accurate orientation determination. Its computational efficiency, especially on GPUs, enables high-speed processing suitable for large mapping experiments. Overall, *LaueMatching* provides a powerful and accessible tool for the materials characterization community. The code implementing this approach is available at https://github.com/AdvancedPhotonSource/LaueMatching.

## Figures and Tables

**Table 1:** Experiment and reconstruction parameters for the different testing and validation cases The refined error for the 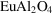 example is the absolute percent error between the refined case (*) and the reference lattice parameter (†) from the literature (see the main text for details). The *a* and *c* values for the lattice parameter were allowed to change while unit-cell volume was fixed.

Sample	Ni	Al	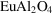 reference (*)	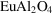 input	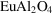 refined (†)	Refined error ( 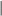 † − * 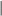 )
*a* (nm)	0.35238	0.40498826	0.844478	0.802254	0.849215	0.5%
*b* (nm)	0.35238	0.40498826	0.882388	0.882388	0.882388	–
*c* (nm)	0.35238	0.40498826	0.515643	0.542781	0.512766	0.56%
α ( 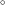 )	90	90	90	90	90	–
β ( 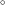 )	90	90	93.1854	93.1854	93.1854	–
γ ( 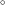 )	90	90	90	90	90	–

**T** (m)	0.028745	0.028720	0.025200	0.025200	0.025200	–
	0.002788	0.003010	−0.002876	−0.002876	−0.002876	–
	0.513115	0.513097	0.510882	0.510882	0.510882	–

**R** (radians)	−1.20131258	−1.20127231	−1.20070188	−1.20070188	−1.20070188	–
	−1.21399082	−1.21381742	−1.21233322	−1.21233322	−1.21233322	–
	−1.21881158	−1.21879073	−1.21883784	−1.21883784	−1.21883784	–
